# Differential Effects of Arsenic in Drinking Water on Mouse Hepatic and Intestinal Heme Oxygenase-1 Expression

**DOI:** 10.3390/antiox11091835

**Published:** 2022-09-18

**Authors:** Hui Li, Xiaoyu Fan, Xiangmeng Wu, Weiguo Han, Mary Kay Amistadi, Pengfei Liu, Donna Zhang, Jon Chorover, Xinxin Ding, Qing-Yu Zhang

**Affiliations:** 1 Department of Pharmacology and Toxicology, College of Pharmacy, University of Arizona, Tucson, AZ 85721, USA; 2 Department of Environmental Science, College of Agriculture and Life Sciences, University of Arizona, Tucson, AZ 85721, USA

**Keywords:** heme oxygenase, arsenic, small intestine, liver

## Abstract

Arsenic exposure has been associated with the risks of various diseases, including cancers and metabolic diseases. The aim of this study was to examine the effects of arsenic exposure via drinking water on the expression of heme oxygenase-1 (HO-1), a major responsive gene to arsenic-induced oxidative stress, in mouse intestinal epithelial cells which is the first site of exposure for ingested arsenic, and the liver, a known target of arsenic toxicity. The expression of HO-1 was determined at mRNA, protein, or enzymic activity levels in mice exposed to sodium arsenite through drinking water, at various doses (0, 2.5, 10, 25, 100 ppm), and for various time periods (1, 3, 7, or 28 days). HO-1 was significantly induced in the intestine, but not liver, at arsenic doses of 25 ppm or lower. The intestinal HO-1 induction was seen in both males and females, plateaued within 1–3 days of exposure, and was accompanied by increases in microsomal HO activity. In mice exposed to 25-ppm of arsenite for 7 days, total arsenic and As(III) levels in intestinal epithelial cells were significantly higher than in the liver. These findings identify intestinal epithelial cells as likely preferential targets for arsenic toxicity and support further studies on the functional consequences of intestinal HO-1 induction.

## 1. Introduction

Arsenic is a naturally occurring toxic and carcinogenic metalloid widely distributed in the upper Earth’s crust, with abundances ranging from 0.1 to over 1000 mg kg^−1^ in soil [1]. Arsenic displays both metallic and nonmetallic features, and it naturally exists in the trivalent (As^III^) and pentavalent (As^V^) forms. Human exposure to arsenic can occur through food, air, and drinking water. Consumption of arsenic-contaminated drinking water has been linked with numerous adverse health outcomes, including cardiovascular diseases, respiratory diseases, neurological diseases, immune system diseases, metabolic disorders, and cancers [2,3,4,5]. Because of the abundant evidence for arsenic toxicity, the inorganic arsenic drinking water standard in the USA was lowered in 2001 from 50 ppb to 10 ppb [6]. However, in some private wells in the United States, arsenic levels in drinking water were reported to be as high as 3100 ppb [7]. In West Bengal of India, arsenic concentrations in tube-well water were between 150 and 200 ppb [8].

The mechanisms of arsenic’s toxic effects are still not fully understood; several modes of action have been suggested, where oxidative stress is commonly involved [9,10,11]. As a defensive response, antioxidant enzymes can be affected by arsenic exposure [12,13]. Nuclear factor erythroid 2-related factor 2 (Nrf2) is a master regulator of the gene expression of antioxidant enzymes [14]. Arsenic has been reported to induce Nrf2 in different tissues, leading to transcriptional upregulation of its downstream target genes, such as glutathione peroxidase, glutathione S-transferases (GSTs), heme oxygenase-1 (HO-1), and NAD(P)H quinone dehydrogenase 1 (NQO1) [15,16,17]. Among Nrf2 targets, HO-1 is a key protein in the response to diseases triggered by oxidative stress [18], and HO-1 induction is considered to be a common response to oxidative stress [19,20]. 

HO-1 as well as its non-inducible isozyme HO-2 are the rate-limiting enzymes in heme metabolism, catalyzing the degradation of heme to biliverdin, CO, and iron. Other than functioning to control the systemic amount of heme, the HOs also play an important role in recycling iron. Moreover, the byproducts of heme degradation, biliverdin and its further metabolite bilirubin, are reported to exert antioxidant and anti-inflammatory effects [18,20]. 

A number of studies have reported HO-1 induction by arsenic exposure [21,22,23,24,25,26,27,28]. The liver is the most extensively studied organ in this regard. However, the effects of arsenic exposure on HO-1 expression in the intestine, the main site of absorption of ingested inorganic arsenic [29], is less well characterized. In the present study, we aim to perform a detailed analysis of the ability of arsenic exposure to induce HO-1 expression in the intestine. Mice were exposed to sodium arsenite in drinking water at various doses, and for different lengths of time, and then intestinal and hepatic HO-1 mRNA and protein expression, as well as microsomal HO activity, were compared between exposed and control (drinking water alone) groups. Total arsenic and arsenic species levels were also determined in the serum, small intestinal epithelial cells, and liver from arsenic-exposed and control mice. Here, we report not only that the expression of HO-1 was induced by arsenic in the intestine at arsenic doses as low as 2.5 ppm in drinking water, but also that the HO-1 induction occurred only in the intestine, but not in the liver, when arsenic doses were 25 ppm or lower in drinking water. The intestinal specific HO-1 induction was observed in both males and females, persisted in all exposure periods tested (1–28 days), and was accompanied by greater accumulation of total arsenic and As(III) in the intestine than in the liver. Our results indicate that the arsenic exposure via drinking water preferentially induces intestinal HO-1 expression, and point to the need to better understand functional consequences of intestinal HO-1 induction in the protective as well as toxic mechanisms associated with arsenic exposure.

## 2. Materials and Methods

### 2.1. Chemicals and Materials

Sodium arsenite (purity ≥ 98%) and *β*-nicotinamide adenine dinucleotide phosphate, reduced form (NADPH) (purity ≥ 97%), were purchased from Sigma-Aldrich (St. Louis, MO, USA). Biliverdin and biliverdin-d4 (purity ≥ 90%) were obtained from Toronto Research Chemicals (North York, ON, Canada). All solvents for high-performance liquid chromatography-mass spectrometry were from Thermo Fisher Scientific (Houston, TX, USA) and of LC-MS grade. 

Halt Protease Inhibitor Cocktail (PI) (100X) was purchased from Thermo Scientific (Rockford, IL, USA). TRIzol Reagent, SuperScript III First-Strand Synthesis System for RT-PCR, NuPAGE LDS sample buffer (4×) and NuPAGE sample reducing agent (10×) were purchased from Invitrogen (Carlsbad, CA, USA). A miRNeasy mini kit (including QIAzol lysis reagent) was from Qiagen (Germantown, MD, USA). SYBR Select Master Mix was from Applied Biosystems (Waltham, MA, USA). Rabbit polyclonal anti-HO-1 was from Enzo Life Sciences (Farmingdale, NY, USA); rabbit anti-calnexin was from Abcam (Cambridge, MA, USA); and peroxidase-conjugated goat anti-rabbit IgG was from Sigma-Aldrich. Bilirubin oxidase (EC 1.3.3.5, from Myrothecium species, ≥0.8 U/mg) and BCA protein assay kit were from Thermo Fisher Scientific (Waltham, MA, USA).

### 2.2. Animals and Treatments 

Male and female C57BL/6 mice (2–4-month-old) were used. Mice were fed a standard chow diet (Envigo 7013) and water ad libitum and were maintained under 12 h on/12 h off light cycles. Mice were given sodium arsenite dissolved in drinking water at various concentrations and for various lengths of time. Mice given water alone were used as the control. All procedures involving mice were approved by the University of Arizona Institutional Animal Care and Use Committee (ethical protocol code: 17-355).

### 2.3. Isolation of Intestinal Epithelial Cells and Preparation of Intestinal and Hepatic Microsomes

Mouse small intestinal epithelial cells were obtained as previously reported by Zhang et al. with slight modifications [30]. In brief, the whole small intestine was excised, cut longitudinally, and washed immediately with ice-cold phosphate-buffered saline (PBS, pH 7.2) to remove contents. The well-washed intestine segment was then placed into an ice-cold buffer B (PBS containing 1.5 mM sodium EDTA, 3 U/mL sodium heparin, 80 μg/mL dithiothreitol, and 40 μg/mL phenylmethanesulfonylfluoride (PMSF)), to loosen mucosal cells. After 15–20 min, the mucosal cells were scrapped, collected, and transferred to 1 mL of ice-cold buffer B, followed by spinning at 8000 g for 1 min. The pelleted cells were stored at −80 °C before use.

Microsomes from mouse small intestine epithelial cells and mouse liver tissue were prepared following previously established methods, with some modifications in the homogenization buffer and storage buffer [30,31]. The following buffers were used: intestinal homogenization buffer, 5 mM histidine, 250 mM sucrose, 0.5 mM sodium EDTA, 40 μg/mL PMSF, and PI cocktail, pH 7.0; intestinal microsomal storage buffer, 0.1 M Tris-HCl, 0.15 M NaCl, 15% glycerol, 0.5 mM PMSF, and PI, pH 7.4; hepatic homogenization buffer, 0.1 M Tris-acetate, 1 mM sodium EDTA, and 0.15 M KCl, pH 7.4; hepatic microsomal storage buffer, 50 mM Tris-acetate, 0.1 mM sodium EDTA, and 20% glycerol, pH 7.4. The total protein concentration of microsomes was determined using a BCA protein assay kit with bovine serum albumin as the standard according to the manufacturer’s protocol.

### 2.4. Total RNA Extraction and Quantitative Real-Time Polymerase Chain Reaction (RT-PCR) Analysis

Total RNA was isolated from mouse liver tissues and mouse small intestinal epithelial cells using TRIzol Reagent and miRNeasy mini kits according to the manufacturers’ protocols, respectively. The concentration and purity of resulting total RNA were determined spectrally using Nanodrop (Thermo Fisher Scientific, Waltham, MA, USA). First-strand cDNA was then synthesized with the extracted total RNA as a template, using the SuperScript III First-Strand Synthesis System, following the manufacturer’s instructions. Quantitative RT-PCR was carried out using a QuantStudio Real-Time PCR System (Applied Biosystems, Foster City, CA, USA) in 96-well plates (MicroAmp Optical 96-Well Reaction Plate, Thermo Fisher Scientific, Waltham, MA, USA). Samples were analyzed in triplicates. The PCR mixtures contained 5 μL of SYBR Select Master Mix, 0.5 μM PCR primers, and first-strand cDNA templates in various amounts, in a total reaction volume of 10 μL. Glyceraldehyde 3-phosphate dehydrogenase (GAPDH) was used to normalize mRNA abundance. Reactions were initiated with a denaturation step at 95 °C for 10 min, followed by 45 cycles of 95 °C for 15 s, 56 °C for 30 s, and 60 °C for 60 s, and a final extension step for 1 min at 60 °C. The fold change of gene expression levels between arsenic-exposed and unexposed control samples was calculated as described previously. Primers used for mouse genes were as follows: glutathione S-transferase mu 1 (GSTM1) (accession number: NM_010358) forward, 5′-cctggatggagagacagagg-3′, and reverse, 5′-gaccttgtcccctgcaaa-3′; NQO1 (accession number: NM_008706) forward, 5′-agggttcggtattacgatcc-3′, and reverse, 5′-agtacaatcagggctcttctcg-3′; HO-1 (accession number: NM_010442) forward, 5′-acactctggagatgacacct-3′, and reverse, 5′-ttgtgttcctctgtcagcatc-3′; GAPDH (accession number: NM_008084) forward, 5′-tgtgaacggatttggccgta-3′, and reverse, 5′-tcgctcctggaagatggtga-3′ [30].

### 2.5. Immunoblot Analysis

Microsomes from mouse small intestinal epithelial cells (5 μg or 10 μg) and liver tissue (5 μg) were heated with an LDS sample buffer and sample reducing agent at 70 °C for 10 min. Proteins were separated on 10% SDS-PAGE gels and then transferred to nitrocellulose membranes (0.45 μm, Bio-Rad Laboratories). After blocking in 5% nonfat dry milk for 1 h at room temperature, the membranes were incubated with the primary antibody (rabbit polyclonal anti-HO-1, 1:1000) at 4 °C overnight. Calnexin (rabbit anti-calnexin, 1:1200) served as the loading control. Peroxidase-conjugated goat anti-rabbit IgG was used as a secondary antibody. Protein bands were visualized with SuperSignal West Femto Maximum Sensitivity substrate (for HO-1 protein determination, Thermo Fisher Scientific, Rockford, IL, USA) or ECL Western blotting detection reagents (for calnexin protein determination, GE Healthcare, Piscataway, NJ, USA), respectively, and imaged using the ChemiDoc XRS1 Imaging System (Bio-Rad Laboratories, Hercules, CA, USA).

### 2.6. In Vitro Assay of HO Activity

The activities of HO protein in intestinal and hepatic microsomes were determined by the method established by Iwamori and coworkers, with some modifications [32]. Briefly, the incubation mixtures contained 0.2 mg microsomal proteins, 25 µM hemin and 0.025 U/mL bilirubin oxidase in 0.05 M potassium phosphate buffer (pH 7.4) containing 1 mM MgCl_2,_ in a final volume of 100 µL. The reactions were initiated by the addition of NADPH to 1.0 mM, after a 3-min pre-incubation. After incubation at 37 °C for 20 min or 0 min (control) in the dark, the reactions were quenched by the addition of 200 µL of ice-cold methanol. The resulting samples were spiked with 10 μL of biliverdin-d_4_ (in methanol, internal standard, final concentration 0.08 µM), vortexed and spun at 12,000 g for 10 min. The supernatant was mixed with 1 mL of water and extracted by solid phase extraction (SPE; C18, Biotage, Charlottesville, VA, USA). Samples were eluted from SPE cartridges with 500 μL of methanol and aliquots were used for liquid chromatography-tandem mass spectrometry (LC-MS/MS) analysis. HO activity was expressed as pmol of biliverdin formed per mg microsomal protein per min.

### 2.7. LC-MS/MS Analysis

Biliverdin and biliverdin-d_4_ were analyzed on an AB-SCIEX model Q-Trap 6500+ mass spectrometer (AB-SCIEX, Framingham, MA, USA) interfaced with an Agilent 1290 Infinity Series ultra-performance liquid chromatography system. Chromatographic separation was carried out on an Agilent ZORBAX Eclipse Plus C18 column (1.8 μm, 2.1 × 50 mm) using gradient elution at a flow rate of 0.2 mL/min for 10 min. The mobile phases were composed of 0.1% (*v*/*v*) formic acid in water (A) and 0.1% (*v*/*v*) formic acid in methanol (B). The linear gradient was as follows: 30% B, 0−1 min; 30−99% B 1–1.1 min; and 99% B 1.1−6.5 min. Biliverdin and biliverdin-d_4_ (retention time 2.7 min) were detected using electrospray ionization in the positive ion mode by multiple reaction monitoring scanning. The mass spectrometer parameters were optimized and set as follows: curtain gas, gas 1, and gas 2 were 30, 25, and 25 psi, respectively; ion source temperature was at 500 °C; and ion spray voltage, declustering potential, entrance potential, and collision cell exit potential were 5500, 90, 10, and 12 V, respectively. The transitions (and corresponding collision energy) were *m/z* 583.1/297.1 (45) for biliverdin and *m/z* 587.3/299.2 (42) for biliverdin-d_4_. All data were analyzed using the AB SCIEX Analyst 1.6.3 software (Applied Biosystems, Framingham, MA, USA).

### 2.8. Quantification of Total Arsenic and Arsenic Species in Serum and Tissues

All samples (serum (~450 µL) and homogenized liver tissue (250–350 mg wet weight) and small intestinal epithelial cells (150–250 mg cell pellet weight)) were digested in 5 mL 0.15-M (~1%) HNO_3_ at room temperature overnight, followed by heating on a Digi-prep HotBlock (SCP Science, Champlain, NY, USA), starting at 40 °C and gradually increasing to 90 °C, for approximately two hours. A certified reference material of fish tissue (DORM-4; https://nrc.canada.ca/en/certifications-evaluations-standards/certified-reference-materials/list/49/html (Last accessed on 19 May 2022) was included to monitor sample recovery for the digestion protocol. Total arsenic levels and arsenic species were measured as described [33,34], at the Arizona Laboratory for Emerging Contaminants (ALEC) of the University of Arizona, using an Agilent 8900 inductively coupled plasma-mass spectrometer (ICP-MS) (Santa Clara, CA, USA). LC separation was performed on a Hamilton PRP-X100 column and guard cartridge (Hamilton Company, Reno, NV, USA), with a gradient of ammonium carbonate mobile phase (10 to 50 mM) at pH 8.6. The solution analyzed for arsenic species was also used to measure the total arsenic using ICP-MS, as described [33,34].

### 2.9. Statistical Analysis

Statistical analysis was performed using the GraphPad Prism 7 software (GraphPad Software, San Diego, CA, USA). Data are expressed as the mean ± SD, and differences were considered significant at *p* < 0.05.

## 3. Results

### 3.1. Effects of Arsenic Exposure via Drinking Water on the Expression of Oxidative Stress Responsive Genes in Mouse Liver and Intestine

**Figure 1:**
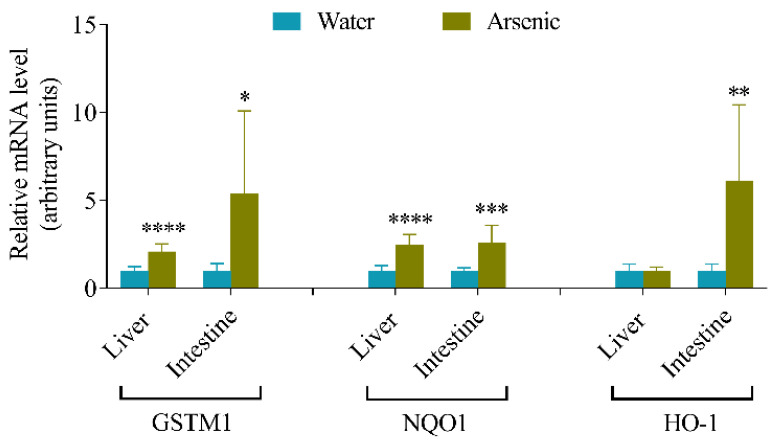
Effects of oral arsenic exposure on the expression of hepatic and intestinal oxidative stress responsive genes. Male mice (2–3-month-old) were exposed to sodium arsenite in drinking water at 25 ppm for 28 days. Control mice were given water alone. Data (means ± S.D.) represent relative mRNA expression levels of GSTM1, NQO1 and HO-1 in mouse liver and small intestinal epithelial cells, normalized by GAPDH mRNA levels. *, *p* < 0.05; **, *p* < 0.01, ***, *p* < 0.001; ****, *p* < 0.0001, vs. the corresponding water control group, *n* = 5–9 (unpaired t-test, two-tailed).

To determine the impact of chronic ingestion of inorganic arsenic on oxidative stress responsive genes, we exposed mice to sodium arsenite in drinking water at 25 ppm for 28 days. The mRNA expression levels of GSTM1, NQO1, and HO-1 in the liver and small intestinal epithelial cells were examined (Figure 1). After the 28-day oral arsenic exposure, the GSTM1 mRNA levels were induced by 2.1- and 5.4-fold in the liver and small intestine, respectively, and the NQO1 mRNA levels were induced by 2.5- and 2.6-fold in the liver and small intestine, respectively, compared to the control mice. However, the HO-1 mRNA induction was only observed in the small intestine by 6.1-fold, compared to the control mice, but not in the liver. The results indicate that the relative mRNA expression levels of GSTM1 and NQO1 were upregulated in both liver and intestine after the arsenic exposure. However, the induction of HO-1 mRNA expression by arsenic exposure occurred only in the small intestine, but not in the liver. 

**Figure 2:**
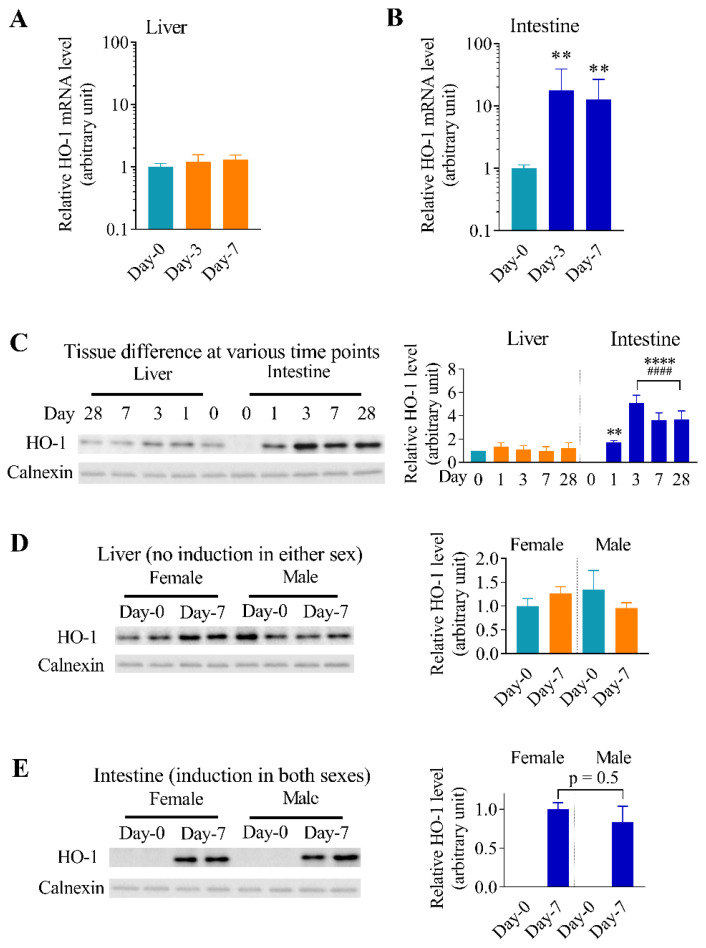
Differential effects of oral arsenic exposure on hepatic and intestinal HO-1 mRNA and protein expression. Mice (3–4-month-old, male or female) were exposed to 25 ppm arsenite via drinking water. Relative HO-1 mRNA levels (means ± S.D.) in the liver (*n* = 3) (**A**) or small intestinal epithelial cells (*n* = 6) (**B**) of male mice, normalized by GAPDH mRNA levels, are shown. **, *p* < 0.01 (Kruskal–Wallis test followed by Dunn’s multiple comparisons test); vs. Day-0. (**C**–**E**) Immunoblot and densitometry analysis of HO-1 protein expression in the liver and small intestine. Microsomes were prepared from pooled small intestinal epithelial cells or liver tissues of 3–4 mice per group. Calnexin was detected and served as the loading control. **C.** Time course of HO-1 protein induction in liver and intestine. Each lane contained 5 µg of hepatic or intestinal microsomal protein. Densitometry data were from three separate blotting experiments. **, *p* < 0.01, ****, *p* < 0.0001, vs. Day-0 intestine; ^####^, *p* < 0.0001 vs. liver of corresponding exposure time (one-way ANOVA followed by Tukey’s multiple comparisons test. (**D**,**E**). Comparing hepatic (**D**) and intestinal (**E**) microsomal HO-1 levels in male and female mice. Each lane contained 5 µg of total protein. Samples were analyzed in duplicates. Densitometry data were normalized by calnexin level in each sample and the levels in female liver Day-0 (**C**,**D**), or female intestine day 7 (**E**) were set to 1.0. Data presented are typical of three separate experiments.

Relative HO-1 mRNA levels were further determined in the liver and small intestinal epithelial cells from mice exposed to 25 ppm of arsenite in drinking water for shorter periods of time (3 or 7 days). No significant changes in the hepatic HO-1 mRNA expression were observed for either of the exposure time points (Figure 2A). However, intestinal HO-1 mRNA levels were increased at both exposure time points tested (Figure 2B).

### 3.2. Differential Effects of Oral Arsenic Exposure on Hepatic and Intestinal HO-1 Protein Expression

To investigate whether there were concomitant changes in HO-1 protein expression after arsenic exposure, HO-1 protein levels were determined in microsomes from liver tissue and small intestinal epithelial cells. As expected, hepatic HO-1 protein levels were not changed in mice after exposure to 25 ppm arsenite in drinking water for up to 28 days; conversely, intestinal HO-1 protein expression was induced as early as one day after commencing exposure to arsenic (Figure 2C–E). Notably, while HO-1 was detected at low amounts in the Day-0 liver microsomes with 5 µg of total protein analyzed, it was not detected in the Day-0 intestine when the same amount of intestinal microsomal proteins was used for the immunoblot analysis (Figure 2C). However, after exposure to arsenic at 25 ppm in drinking water, HO-1 protein levels in the intestine were much higher than those in the liver. The lack of HO-1 protein induction in the liver and the robust induction of HO-1 protein in the intestine were confirmed in both male and female mice, as demonstrated in Figure 2D,E for mice exposed to arsenic for 7 days. The extent of HO-1 protein induction in the intestinal epithelial cells was similar between males and females (*p* = 0.5).

### 3.3. Differential Effects of Oral Arsenic Exposure on Hepatic and Intestinal HO Activity

**Figure 3:**
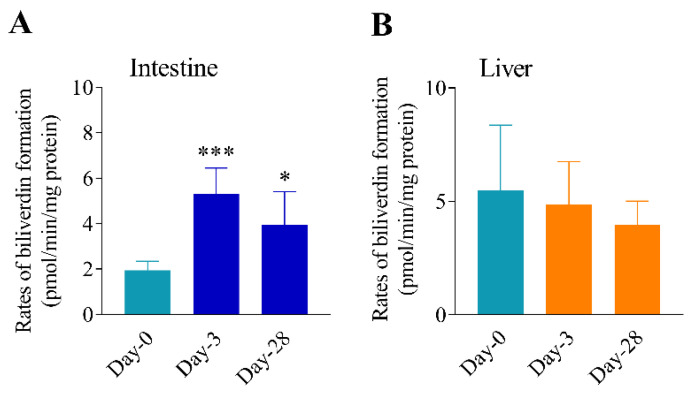
Differential effects of oral arsenic exposure on intestinal and hepatic HO enzymatic activity. Mice (3–4-month-old, female) were exposed to 25 ppm arsenite in drinking water for 0, 3, or 28 days. Rates of metabolism of hemin (to biliverdin) were determined for small intestinal (**A**) or hepatic (**B**) microsomal preparations. Microsomes were prepared from small intestinal epithelial cells or liver tissue from individual mice. Reaction mixtures contained 2.0 mg/mL microsomal proteins, 25 µM hemin, 1.0 mM NADPH, and 0.025 U/mL bilirubin oxidase. Data represent means ± S.D. (*n* = 4–7) *, *p* < 0.05; ***, *p* < 0.001, vs. control (Day-0) group (one-way ANOVA followed by Tukey’s multiple comparisons test).

HO enzyme activities in the liver and intestine were further evaluated. Figure 3 shows rates of heme metabolism by the liver and intestinal microsomes from mice exposed to 25 ppm arsenite in drinking water for various periods of time. Intestinal HO activity was significantly increased after 3 or 28 days of exposure to arsenic, compared to Day 0 controls (Figure 3A); there was no significant difference in enzyme activity between Day 3 and Day 28. The two time points were selected to represent acute (Day 3) and persistent (Day 28) changes. The maximal HO activity induction was 2.7-fold (on Day 3). Conversely, consistent with the lack of induction of hepatic HO-1 mRNA and protein, a significant increase in hepatic HO activity was not observed when comparing microsomes from mice exposed to arsenic for 0, 3, or 28 days (Figure 3B). Notably, in unexposed (Day 0) mice, the constitutive level of HO activity in intestinal epithelial cells was less than one half of the hepatic microsomal HO activity (1.9 vs. 5.5); whereas, after exposure to arsenic, intestinal HO activity was comparable to hepatic HO activity.

### 3.4. Tissue Arsenic Levels in Mice Exposed to Arsenic in Drinking Water

**Figure 4:**
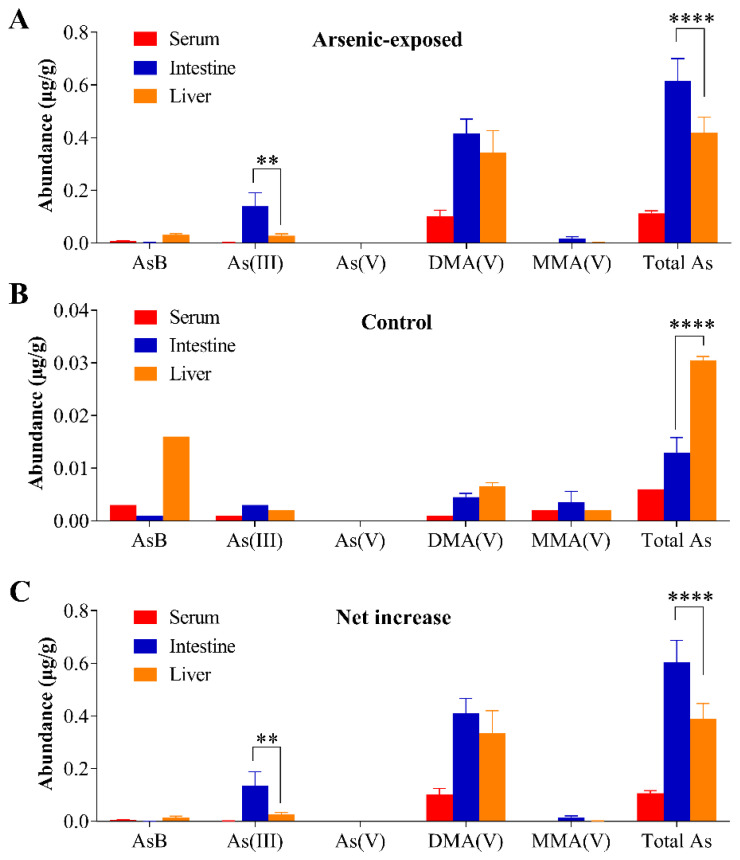
Arsenic levels in tissues from control mice and mice exposed to 25-ppm arsenite orally. Total arsenic and arsenic species levels were determined in the serum, small intestinal epithelial cells, and liver from mice (3–4-month-old female) exposed to 25-ppm arsenite in drinking water for 7 days (**A**), or with drinking water alone (**B**). Net changes in arsenic levels resulting from drinking arsenic water were calculated and shown in panel (**C**) Data represent means ± S.D.; **, *p* < 0.01; ****, *p* < 0.0001 (two-way ANOVA followed by Sidak’s multiple comparisons test). Each serum sample was pooled from 2 mice (*n* = 1 for control and 2 for exposed); intestinal epithelial cells or liver tissues from individual mice (*n* = 2 for control and 4 for exposed) were analyzed. Abbreviations: AsB, arsenobetaine; As(III), arsenite; As(V), arsenate; DMA(V), dimethylarsinic acid; and MMA(V), monomethylarsonic acid.

Figure 4 shows total arsenic and arsenic species levels in the liver tissue, intestinal epithelial cells, and serum from unexposed mice and mice exposed to 25 ppm of arsenite in drinking water for 7 days. Total arsenic and As(III) concentrations in the intestine were 47% and 4.1-fold, respectively, higher than those seen in the liver after the arsenic exposure (Figure 4A). In addition, dimethylarsinic acid (DMA(V)) was found to be the major metabolite in all tissues studied, accounting for 82% in the liver, 68% in the intestine, and 90% in the serum, compared to the corresponding total arsenic levels (Figure 4A). The other arsenic species detected, including As(V), MMA(V), and AsB, were all at relatively low levels in all three tissues.

Notably, all of these arsenic species were also detected in unexposed mice, though at very low levels. Background concentrations of total arsenic and arsenite (As(III)) in all samples tested were less than 0.05 µg/g sample and 0.004 µg/g sample, respectively (Figure 4B). Contrary to the 25 ppm arsenite-exposed mice, total arsenic levels in the liver of unexposed mice were 2.3-fold higher than in intestine, and the major hepatic arsenic species in these mice was AsB, which accounted for ~53% of total arsenic in liver.

### 3.5. Inducibility of Intestinal HO-1 Expression at Lower Arsenic Doses

**Figure 5:**
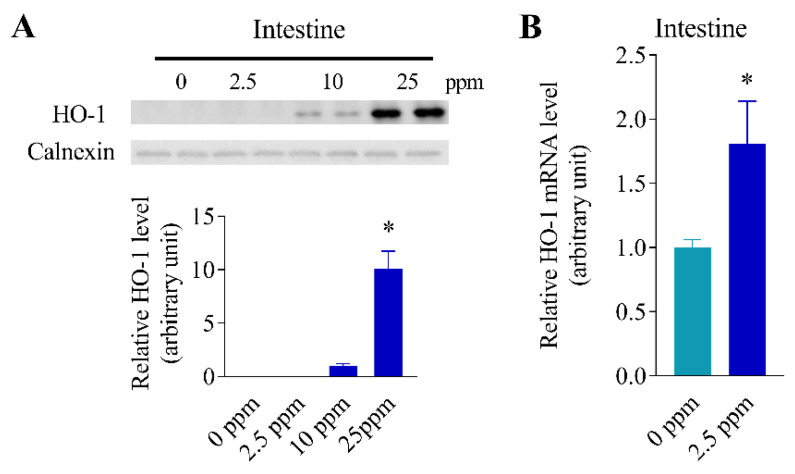
Inducibility of intestinal HO-1 expression at lower arsenic doses. Relative HO-1 protein and mRNA levels were determined for small intestinal epithelial cells of female, 3–4-month-old mice, after 7 days of arsenic exposure via drinking water at 2.5, 10, or 25 ppm. Mice given drinking water only (0 ppm) were used as controls. (**A**) Microsomal HO-1 protein levels were determined by immunoblot analysis. Microsomes were prepared from pooled small intestinal epithelial cells of 3–4 mice per group. Each lane contained 10 µg of total microsomal protein. Samples were analyzed in duplicates. Densitometry data (means ± SD of duplicate determinations) were normalized by calnexin level in each sample and the average level in the 10-ppm sample was set to 1.0. *, *p* < 0.05, vs. 10 ppm (unpaired t-test, two-tailed). (**B**) HO-1 mRNA levels were determined by RT-PCR. Data represent means ± SD of individual mice analyzed (*n* = 3), and were normalized to GAPDH levels; *, *p* < 0.05, vs. 0-ppm control (unpaired t-test, two-tailed).

To examine the ability of lower doses of arsenic to induce HO-1 expression in the intestine, mice were given arsenic at various doses (0, 2.5, 10 or, 25 ppm) in drinking water for 7 days. Immunoblot analyses of intestinal epithelial cell microsomal proteins revealed induction of HO-1 protein by 10 ppm arsenite, but not by 2.5 ppm of arsenite (Figure 5A). However, HO-1 induction by 2.5 ppm of arsenite was demonstrated at the mRNA level (Figure 5B).

### 3.6. Differential Inducibility of Hepatic and Intestinal HO-1 Expression at a Higher Arsenic Dose

**Figure 6:**
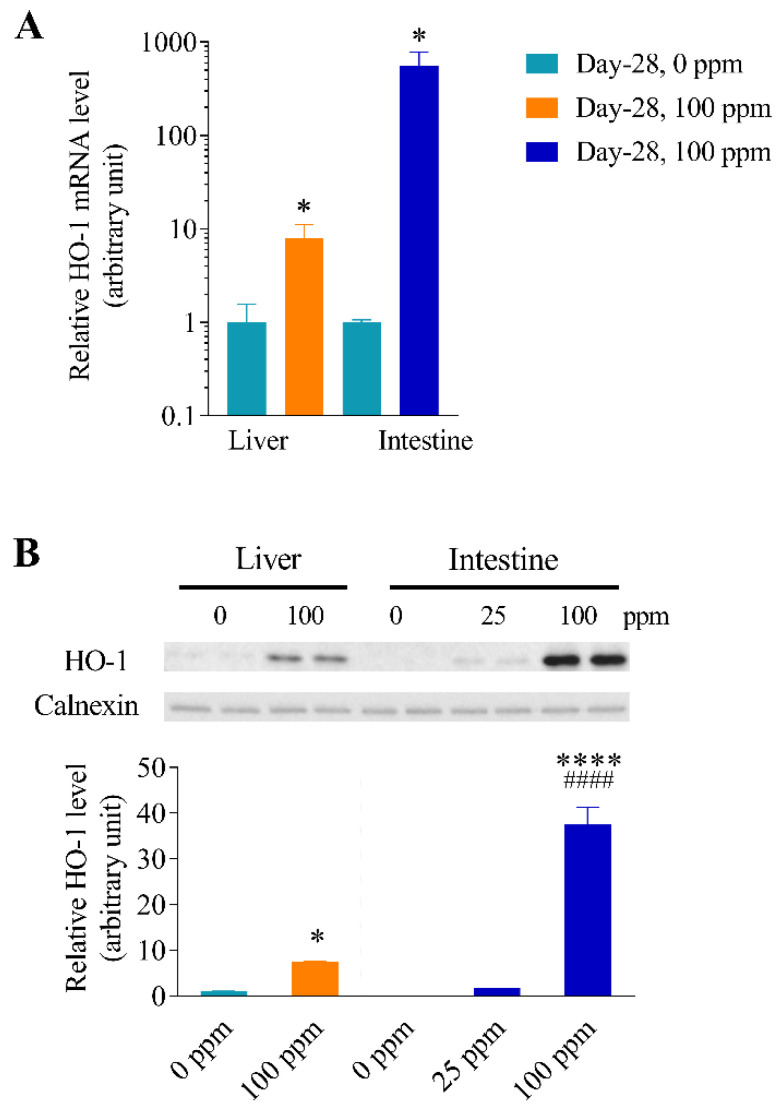
Differential inducibility of hepatic and intestinal HO-1 expression at a higher arsenic dose. Relative HO-1 mRNA (**A**) and protein (**B**) levels were determined for the liver and small intestinal epithelial cells of male, 3–4-month-old mice, after 28 days of arsenic exposure via drinking water. Mice given drinking water alone (0 ppm) were used as control. (**A**). HO-1 mRNA levels determined by RT-PCR. Data represent means ± SD of individual mice analyzed, and were normalized to GAPDH levels; *, *p* < 0.05, 100-ppm (*n* = 4) vs. corresponding 0-ppm control (*n* = 2), unpaired t-test, two-tailed. (**B**). HO-1 protein levels determined by immunoblot analysis. Microsomes were prepared from pooled liver or small intestinal epithelial cells of three mice per group. Each lane contained 5 µg of total protein. Samples were analyzed in duplicates. Densitometry data (means ± SD of duplicate determinations) were normalized by calnexin level in each sample and the average level in the 0-ppm liver sample was set to 1.0. *, *p* < 0.05, ****, *p* < 0.0001, vs. corresponding 0-ppm group; ^####^, *p* < 0.0001, vs. liver 100-ppm group (one-way ANOVA followed by Sidak’s multiple comparisons test).

To determine whether the preferential induction of intestinal HO-1 expression over hepatic HO-1 expression by arsenic in drinking water persisted under conditions that allow induction of hepatic HO-1, mice were exposed to arsenic at 100 ppm for 28 days via drinking water. As shown in Figure 6, hepatic HO-1 expression was induced under these conditions, with increases of 7.9-fold at the mRNA levels (Figure 6A) and 7.5-fold at the protein levels (Figure 6B). However, the extents of induction in the intestinal epithelial cells for HO-1 mRNA (560 fold) and microsomal HO-1 protein (38 fold) were both greater than in the liver. The HO-1 protein level was 5-fold higher in the intestinal epithelial cells than in the liver in 100-ppm arsenite exposed mice (*p* < 0.0001). 

## 4. Discussion

Arsenic-contaminated drinking water poses serious threats to human health. Arsenic dissolved in drinking water can be well absorbed through the gastrointestinal tract. The tissue-selective induction of intestinal HO-1 by arsenic in drinking water (2.5–25 ppm) is a novel finding. Few previous studies have examined the effects of drinking arsenic water on the HO-1 expression in intestinal epithelial cells. However, the lack of induction of hepatic HO-1 expression by a 30-day exposure to 5 ppm inorganic arsenic in drinking water was recently reported [28]; the HO-1 expression in the small intestine was not examined in that study. 

Interestingly, in mice exposed to arsenic at 25 ppm for 28 days, two other oxidative stress sensor genes, GSTM1 and NQO1, were upregulated in both the liver and small intestine (Figure 1), a finding suggesting that GSTM1 and NQO1 are more sensitive markers of arsenic-induced antioxidant response than HO-1 is in the liver. Notably, hepatic HO-1 expression can be induced at higher arsenic levels (100 ppm) in drinking water (Figure 6), though the extent of induction, as well as the induced level of microsomal HO-1 protein, was still higher in the intestine than in the liver under these conditions. 

The arsenic doses of 2.5–25 ppm in drinking water are still quite high compared to the 10 ppb safety standard for drinking water in the USA [6]; but the 2.5 ppm level is close to the ~ 3 ppm arsenite level reported in heavily contaminated well water [7]. Moreover, given the known species differences in rates of arsenic metabolism between mice and humans [35], the 2.5–25 ppm dose range is considered relevant to cases of human exposure in arsenic polluted areas of Bangladesh, on the basis of comparable arsenic levels detected in the liver of exposed people from that region [36], and in the liver of mice exposed to 50 ppm arsenite in drinking water [37].

Arsenic can induce HO-1 in multiple tissues. However, the present study is focused on HO-1 expression in the intestinal epithelial cells, the site of arsenic absorption. It remains to be determined whether the intestinal epithelial cells are more or less sensitive in their response to arsenic exposure than internal organs other than the liver, such as the kidney cortex, where robust HO-1 induction was reported for mice drinking 5 ppm arsenite for 30 days [28]. 

It is yet unclear why the intestine is apparently more sensitive than the liver in HO-1 induction by arsenic in drinking water. Given that the small intestine is the portal-of-entry organ for ingested arsenic, we speculated that arsenic levels in the intestine may be higher than in the liver, a scenario that would support preferential induction of HO-1 expression in the intestine. This hypothesis appears to be valid, according to arsenic levels measured for mice exposed to 25 ppm arsenite for 7-day concentrations of total arsenic and As(III), the most toxic inorganic form [38], were significantly higher in the intestine than in the liver (Figure 4A,C). It can also be rationalized that a dose-related further increase in hepatic arsenic level, when mice drank water containing 100 ppm of arsenite, led to the observed induction of hepatic HO-1 (Figure 6). Notably, the observation of a much lower ratio of As(III) to DMA(V), an arsenic metabolite that is less toxic than As(III) [39], in the liver (0.08) than in the intestine (0.34) seems to suggest a lower ability of the intestine to metabolize As(III) (Figure 4A). 

Basal levels of HO-1 protein in the liver were higher than those in the intestinal epithelial cells, where HO-1 was not detected by immunoblotting (Figure 2C). This “constitutive” HO-1 expression in the liver, which may be due to basal Nrf2 activation by the low levels of accumulated arsenic derived from the normal chow diet and drinking water, was consistent with the higher total arsenic level in the liver than in intestinal epithelial cells (Figure 4B). In that regard, the basal expression levels of Nrf2 protein in various mouse organs are expected to be very low, as Nrf2 is constitutively targeted for Keap1-mediated degradation [40]. Thus, it remains to be determined whether liver and intestinal epithelial cells differ in their basal Nrf2 expression levels and whether such a difference would account for the observed differences in the HO-1 expression level. Alternatively, hepatic HO-1-basal expression may be activated by transcription factors other than Nrf2. However, after exposure to arsenic at 25 ppm in drinking water, the intestinal HO-1 protein levels surpassed those in the liver (Figure 2C). 

The induction of intestinal HO-1 expression by arsenic in drinking water has some interesting features. The induction occurred in both males and females, with no difference in extent of induction (Figure 2E). Induction was detectable at just one day after exposure and persisted with continuous exposure (Figure 2B,C). The induction appeared to be dose-responsive at both mRNA and protein levels within the dose range of 2.5–25 ppm, showing greater induction at higher dose (Figure 2, Figure 5, and Figure 6); but the induced levels did not continue to increase beyond day 1 (mRNA) or day 3 (protein), which suggested that the ingested arsenic did not accumulate in the intestinal epithelial cells under the conditions examined (with up to 25 ppm arsenite in drinking water). However, further arsenic measurement studies are needed to confirm this deduction, particularly for animals exposed to arsenic for longer durations, beyond the 28 days tested here. 

The induction of intestinal HO-1 protein was accompanied by increases in microsomal HO activity toward the heme metabolism (Figure 3). Notably, contrary to the large increases in HO-1 mRNA and protein levels, arsenic exposure only led to a less-than-3-fold increase in the rates of microsomal heme metabolism. This finding is not surprising, as, besides HO-1, the constitutively expressed HO-2 also has significant activities toward heme metabolism [20]. HO-2 protein level was reported to be much higher than basal HO-1 levels in the mouse intestine [41], though HO-2 appeared to be less efficient, with ~10-fold lower Vmax and ~ 2-fold higher Km values than those of HO-1 [42,43,44]. Thus, the contribution of HO-2 to microsomal HO activity would mask the induction of HO-1 activity. Additionally, the level of P450 oxidoreductase may also be a limiting factor for HO activity in intestinal microsomes, as was reported for the testis [45]. 

Oxidative stress is a well-accepted mechanism of arsenic-mediated toxicity. The preferential induction of HO-1 in the intestine suggests that the intestinal epithelial cells are under greater oxidative stress than the liver when mice were exposed to arsenic through drinking water. Thus, greater attention should be paid to potential toxicity of arsenic in the intestine, particularly following chronic exposure. Furthermore, the increase in HO activity may directly contribute to the protective mechanism against arsenic-induced oxidative stress, though chronic hyperactivation of heme metabolism may also lead to adverse effects [18,43,46]. 

The in vivo significance of the intestinal HO-1 induction in the context of arsenic exposure remains to be defined. Further studies of the effects of arsenic exposure on the homeostasis of heme and heme metabolites are warranted. Additionally, given the known function of HO-1 in regulating mitochondrial quality control in several other organs, such as the heart [47], it will be interesting to determine the effects of arsenic exposure and intestinal HO-1 ablation on mitochondrial quality control in intestinal epithelial cells.

## 5. Conclusions

In conclusion, arsenic in drinking water significantly induces HO-1 expression in mouse intestinal epithelial cells under conditions that do not induce HO-1 expression in the liver. The intestinal HO-1 induction was seen in both males and females, plateaued within 1–3 days of exposure, and was accompanied by increases in microsomal HO activity. This tissue-specific HO-1 induction appears to be due to a slower metabolism of arsenic, which led to higher tissue levels of As(III) and total arsenic, in intestinal epithelial cells than in the liver. These findings identify intestinal epithelial cells as likely preferential targets for arsenic toxicity and support further studies on the functional consequences of intestinal HO-1 induction in the protective mechanisms against arsenic toxicity.

## Data Availability

The data is contained within the article.
